# Oxidative Stress and Cell Senescence Process

**DOI:** 10.3390/antiox11091718

**Published:** 2022-08-30

**Authors:** Raffaella Faraonio

**Affiliations:** Department of Molecular Medicine and Medical Biotechnologies, University of Naples Federico II, 80131 Naples, Italy; raffaella.faraonio@unina.it; Tel.: +39-0817463642

Oxidative stress due to excessive amounts of reactive oxygen species (ROS) and reactive nitrogen species (RNS) plays a leading role in damages to macromolecules and, as such, it represents a key driver of numerous physio-pathological events, including cellular senescence [1]. Even if cell senescence takes part in important physiological processes, like embryonic morphogenesis, tissue homeostasis and repair as well as tumor suppression [2,3,4,5], this phenomenon is increasingly considered a ruling contributor in aging and age-related pathologies [6]. The accumulation of senescent cells can indeed reduce the functionality of organs and limit proper tissue renewal in vivo. For this reason, particular attention is given to strategies that can delay or counteract senescence induced by ROS/RNS deregulation, thus improving healthy aging and mitigating age-related diseases.

In this Special Issue, original research papers provide evidence that oxidative stress-induced senescence could be restricted or even delayed by using different types of synthetic molecules or antioxidants treatments. This will contribute to expanding our knowledge on the potential use of drugs or supplementations that may help modulate senescence with possible therapeutic purposes while avoiding cell damage.

Mounting evidence shows that the senescence of mesenchymal stem cells (MSCs) hinders their potential in regenerative medicine and in stem cell therapy [7]. This is consistent with the observation that senescence occurs in both mitotic and post-mitotic cells as well as in the adult stem cell pools. The original research published in this Special Issue by Wang et al. [8] focuses on metabolic senescence induced in Bone Marrow derived Mesenchymal Stem Cells (BMSCs) through d-galactose (d-gal) treatment. d-gal promotes cell senescence through ROS generation, in particular H_2_O_2_, and it is a widely accepted aging model [9]. By using this model, the authors demonstrated that treatments with exogenous NAD+ protects BMSCs from d-gal-induced senescence and reduces ROS levels in senescent BMSCs. This is in line with the observation that NAD+ levels decrease with aging/senescence [10]. Here, the authors also showed that protective NAD+ effects are causally related to Sirt1 signaling, this being enzyme a NAD+-dependent deacetylase. This study extends previous findings on NAD+ repletion as a possible therapeutic tool to improve mesenchymal stem cell performance along with health or lifespan.

Ok and collaborators explored the effects of FK866, a non-competitive inhibitor of Visfatin (also termed pre-B-cell colony-enhancing factor/nicotinamide phosphoribosyltransferase-Nampt) on premature senescence induced by H_2_O_2_ in human Dental Pulp Cells (hDPCs) [11]. Visfatin is an adipocyte hormone with many physiological and pathological actions, ranging from inflammatory to oxidative stress response/aging [12]. In this original contribution, the authors demonstrated that treatment with FK866 mitigates H_2_O_2_-induced premature senescence and reduces telomere damage in hDPCs. The accurate analysis of FK866 effects demonstrates that this small molecule prevents NADPH consumption and ROS production as well as reducing the expression of SASP factors (IL-1α, IL-6, IL-8, COX-2, and TNF-α) through the abrogation of the NF-κB signaling, the main pathway implicated in SASP production during senescence [13].

Autophagy is a quality control pathway that degrades damaged structures/organelle within cells and captures the resulting molecules for recycling and energy production. This process can be stimulated by oxidative stress and a decrease of autophagy has been related to aging [14]. In fact, the activation of the autophagic process has been shown to prevent oxidative-stress-induced senescence. The original contribution of Maharajan et al. examined the role of the monoterpenoid camphorquinone (CQ) on oxidative-stress-induced senescence in both cellular and in vivo experimental models **[15]**. Here, they used human Bone Marrow Mesenchymal Stem Cells (hBM-MSCs) exposed to H_2_O_2_ and heart tissue taken from d-Gal-aged mice, as heart and brain are the most affected organs by d-Gal treatments. The authors found that CQ administration alleviate senescence markers (SA-beta-Gal, p53, p16, and p21) in both hBM-MSCs and in mouse heart tissue. Focusing their attention on AMPK/SIRT1 and autophagy mechanisms, they provide evidence that the biological benefits of CQ are mediated by the activation of AMPK/SIRT1 signaling, thus promoting autophagy.

Kim et al., in their original contribution, looked at the inhibition of autophagy in the H_2_O_2_-induced senescence of skin fibroblasts, a process mediated by ARG2 (arginase 2) increase [16]. ARG2 is a mitochondrial enzyme that through non-canonical actions induces senescence by reducing autophagy in vascular endothelial cells and keratinocytes [17]. Here, the authors found that ARG2 knockdown inhibited H_2_O_2_-induced fibroblast senescence, whereas ARG2 overexpression promoted it. In line with this, the forced expression of miR-1299, which targets ARG2 3′UTR mRNA, counteracts senescence provoked by H_2_O_2_ treatment. Using the yeast two-hybrid assay, the authors also found that ARG2 interacts with ARL1, a positive regulator of autophagy in yeast, thus revealing a functional link between these two molecules in the human context. In fact, the pro-resolving lipid RvD1 inhibits H_2_O_2_-induced senescence by activating autophagy via ARL1 and downregulating ARG2. This study indicates that the inhibition of ARG2 through miR-1299 and/or RvD1 could be a novel mechanism to interfere with senescence process.

As previously mentioned, redox state and senescence are strictly linked, and thus supplementation with antioxidant molecules is of major interest to counteract the pathophysiology of aging and age-related diseases. In this regard, Varesi et al. provide a comprehensive review on the specific aspects of the main antioxidant molecules capable of counteracting senescence/aging **[18]**. After giving a brief description of the major pathways driving senescence through ROS/RNS deregulation, the authors review and discuss: (a) the role of specific antioxidant enzymes; (b) the potential benefits of mitochondria-targeting compounds (e.g., Coenzyme Q10, SkQs and methylene blue); (c) the protective actions of vitamins, carotenoids, organosulfur compounds (e.g., glutathione) and nitrogen non-protein molecules; (d) the preclinical studies on flavonoids as anti-aging compounds; (e) the effects of non-flavonoids treatments; (f) the importance of specific minerals in the modulation of oxidative stress-induced senescence. Since it is well established that most antioxidants can exert a dual effect (both pro-oxidant and antioxidant), mainly related to the doses used, the authors also underline the importance of examining the optimal dosage for their anti-aging potential as well as the need for further research to find better correlations in vivo.

In this Special Issue, we present a review that brings together important studies on three classes of non-coding RNAs (microRNAs, long non-coding RNAs, and circular RNAs) in relation to the redox control of cell senescence [19]. Non-coding RNAs are endogenous molecules that govern gene regulatory networks, thus impacting both physiological and pathological events, including those evoked by oxidative stress [20,21]. After describing the most important ROS/RNS sources, antioxidant systems, and redox-sensitive signaling pathways driving senescence, we provide a summary of the literature evaluating the activities of these regulatory molecules in the signaling cascades of oxidative stress-induced senescence. Molecular mechanisms mediating the actions of microRNA, long non-coding RNA, and circular RNA in the major redox signaling pathways as well as their regulatory integrated networks are discussed.

I would like to acknowledge the authors that have contributed to this Special Issue “Oxidative Stress and Cell Senescence Process”. In this Special Issue, the contributors highlighted that oxidative stress-induced senescence can be restricted or even delayed by using novel interventions/molecules.
